# Left ventricular mass by cardiac magnetic resonance imaging and adverse cardiovascular outcomes in patients treated with anthracycline-based chemotherapy

**DOI:** 10.1186/1532-429X-14-S1-O30

**Published:** 2012-02-01

**Authors:** Tomas G Neilan, Diego Pena-Herrera, Otavio R Coelho-Filho, Michael Jerosch-Herold, Javid Moslehi, Raymond Kwong

**Affiliations:** 1 Medicine, Massachusetts General Hospital, Boston, MA, USA; 2 Medicine, Brigham and Women's Hospital, Boston, MA, USA; 3 Radiology, Brigham and Women's Hospital, Boston, MA, USA

## Summary

LV mass by CMR is a powerful predictor of adverse cardiovascular outcomes in patients treated with anthracyclines.

## Background

Late gadolinium enhancement (LGE) is a predictor of adverse outcomes in patients. However, limited data exist on the role of LGE, the characteristic CMR findings, and the prognostic variables in patients who develop a cardiomyopathy after treatment with anthracyclines.

## Methods

LGE-CMR imaging was performed in patients with stage B and C heart failure after anthracycline-based chemotherapy. We assessed the association between CMR, EKG, echocardiographic, serum, and clinical variables with adverse outcomes (cardiovascular death and admission for heart failure).

## Results

**Figure 1:**
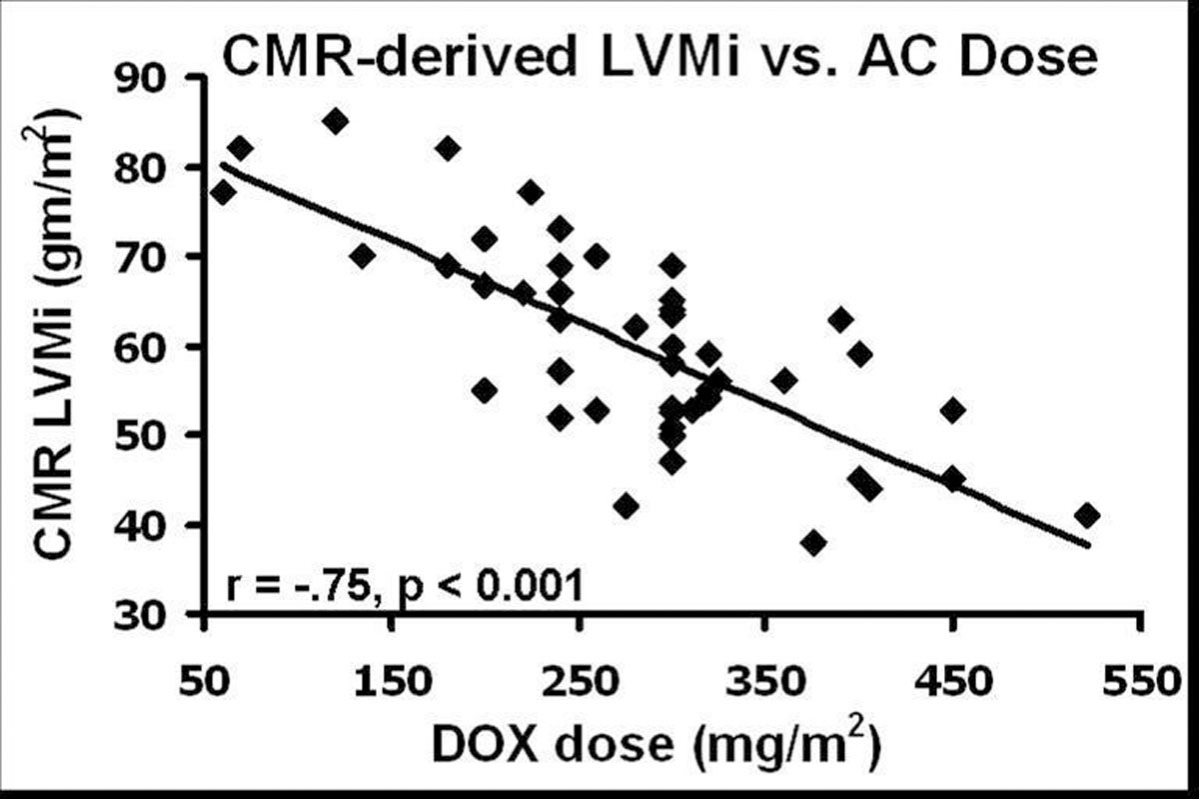


**Figure 2:**
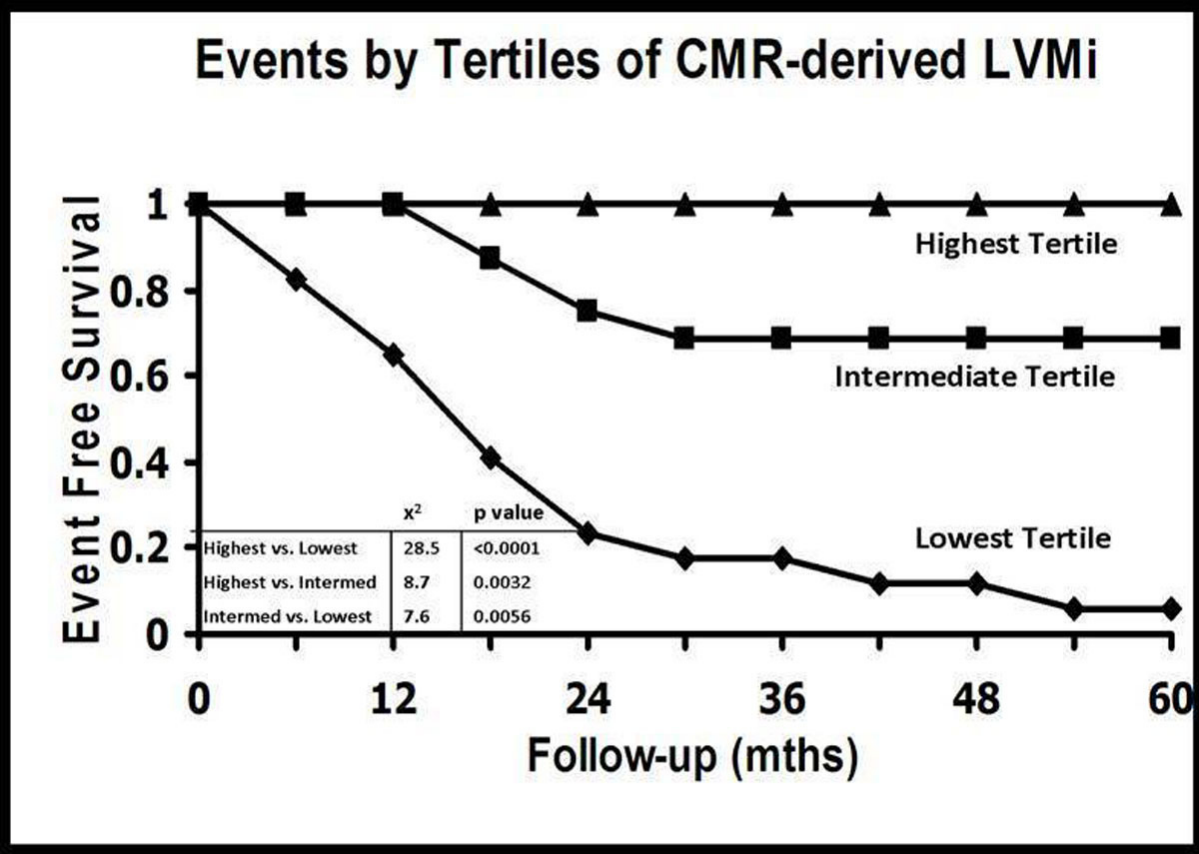


We performed a clinically-indicated CMR study on 50 patients (52% male, mean age of 49 ± 16 years, anthracycline dose of 286 ± 89 mg/m2, and ejection fraction of 38 ± 9%) with AC-mediated cardiomyopathy. Patients presented a median of 45 months after chemotherapy and were followed for a median period of 28 months. LGE was an uncommon finding (3 patients, 6%). There was a strong inverse association between anthracycline dose and indexed left ventricular mass by CMR (r = -.75, p < 0.001, Figure 1). In univariate analysis, indexed LV-mass by CMR demonstrated the strongest unadjusted association with adverse events (hazard ratio: 0.75, chi-squared 26.2, p < 0.001). In a multivariable model, indexed LV-mass demonstrated the strongest association with the primary outcome (Figure 2).

## Conclusions

Residual LV-mass measured by CMR is a powerful predictor of subsequent adverse cardiovascular events in patients with anthracycline-induced cardiotoxicity.

## Funding

Dr. Neilan is supported by an NIH T32 Training Grant (T32HL09430101A1).

